# Mechanical Evaluation of Commercially Available Fibrin Sealants for Cartilage Repair

**DOI:** 10.1177/19476035231163273

**Published:** 2023-03-27

**Authors:** Arya Amirhekmat, Wendy E. Brown, Evelia Y. Salinas, Jerry C. Hu, Kyriacos A. Athanasiou, Dean Wang

**Affiliations:** 1 School of Medicine, University of California, Irvine, Irvine, CA, USA; 2 Department of Orthopaedic Surgery, University of California, Irvine, Orange, CA, USA; 3 Department of Biomedical Engineering, University of California, Irvine, Irvine, CA, USA

**Keywords:** cartilage, fibrin sealant, fibrin glue, Tisseel, Vistaseal

## Abstract

**Objective:**

Fibrin sealants are routinely used for intra-articular surgical fixation of cartilage fragments and implants. However, the mechanical properties of fibrin sealants in the context of cartilage repair are unknown. The purpose of this study was to characterize the adhesive and frictional properties of fibrin sealants using an *ex vivo* model.

**Design:**

Native bovine cartilage-bone composites were assembled with a single application of Tisseel or Vistaseal. Composites were tested in tension and lap shear. In addition, the coefficient of friction (COF) was measured in a native cartilage annulus model alone and with minced cartilage. Finally, the effect of a double application of fibrin sealant was evaluated.

**Results:**

There were no significant differences in tensile modulus, ultimate tensile strength (UTS), shear modulus, or ultimate shear strength (USS) between the 2 fibrin sealants. Both fibrin sealants demonstrated a UTS and USS of <8 and <30 kPa, respectively. There were no differences in COF between the sealants when tested alone or with minced cartilage. A double application of fibrin sealant did not alter the mechanical properties compared with a single application of fibrin sealant.

**Conclusions:**

Fibrin sealant adhesive properties are not affected by the sealant type studied or the number of applications in a bovine cartilage-bone model. Fibrin sealant tribological properties are not affected by sealant type or the addition of minced cartilage. The adhesive properties of Tisseel and Vistaseal were less than those desired for the *in vivo* fixation of cartilage repair implants. These findings motivate the development of an improved cartilage-specific adhesive for cartilage repair applications.

## Introduction

Fibrin sealant is a widely available surgical product that uses fibrinogen and thrombin to create a clot at the location of application.^1^ Commercially available fibrin sealants contain varying amounts of pooled human fibrinogen, thrombin, anti-fibrinolytics, calcium source, and preservatives. Upon application, the fibrinogen and thrombin components are mixed, leading to cleavage of fibrinogen by thrombin to become fibrin. In a manner similar to physiological clotting, a semirigid clot is formed by monomer crosslinking.^2^ Although fibrin sealant is indicated for adjunct hemostasis, it is also commonly used for the surgical fixation of tissues, including skin, nerves, liver, pancreas, pterygium, cartilage, and dura.^3,4^

Within the field of orthopedic surgery, fibrin sealant is commonly used as a fixative for cartilage repair procedures. These include fixation of displaced chondral fragments^5,6^ and cartilage implants, including matrix-induced autologous chondrocyte implantation (MACI) and processed allografts,^7-9^ to the underlying bony base of the defect being treated. For particulated cartilage grafts such as DeNovo NT (Zimmer) and BioCartilage (Arthrex), fibrin sealant is used to both suspend and fixate these grafts.^10-12^ Tissue-engineering-based cartilage repair products currently under development, such as Biocart II (ProChon Biotech, Woburn, MA), Bioseed-C (BioTissue SA, Freiburg, Germany), and Cartipatch (TBF Genie Tissulaire, Lyon, France), also rely on the use of fibrin sealant for implant fixation.^13,14^ Furthermore, fibrin sealants are thought to provide a matrix for cells to migrate into, encouraging long-term implant integration.^15^ While not its intended indication, fibrin sealant is heavily relied upon as a fixative for current and future cartilage repair techniques. However, few studies have examined the adhesive and frictional properties of fibrin sealants in the context of cartilage repair, specifically its adhesiveness to articular cartilage and subchondral bone.

Motivated by the lack of data regarding the mechanical properties of commercially available fibrin sealants for cartilage repair applications, the primary objective of this study was to characterize the adhesive and frictional properties of 2 commonly used commercial fibrin sealants: Tisseel (Baxter Healthcare Corp, Deerfield, IL) and Vistaseal (Ethicon, Raritan, NJ). While both fibrin sealants contain fibrinogen and thrombin, the amount of fibrinogen and the addition of other active and inactive ingredients vary between products. Tisseel contains 67-106 mg/ml of fibrinogen, 2,250-3,750 KIU/ml of synthetic aprotinin, 400-625 units/ml of thrombin, 36-44 μmol/ml of calcium chloride, and other ingredients, including human albumin, sodium chloride, tri-sodium citrate, histidine, niacinamide, polysorbate 80, and water.^16^ Vistaseal contains 80 mg/ml of fibrinogen, 500 IU/ml of thrombin, and other ingredients, including human albumin, sodium citrate, calcium, chloride, sodium chloride, arginine, glycine, l-isoleucine, l-glutamic acid monosodium, and water.^17^ Because fibrin sealant fixation of cartilage fragments and implants is primarily dependent on adhesion to the subchondral bone base of the chondral defect, an *ex vivo* native cartilage-bone composite model was used, and resistance to both tension and shear was tested. In addition, the frictional properties of fibrin sealant itself, as well as minced native cartilage plus fibrin, were compared in an *ex vivo* cartilage defect model. The secondary objectives were to determine whether there are differences in those properties based on fibrin sealant type or number of applications. Double applications of fibrin sealant are clinically performed in scenarios when a bottom layer of fibrin sealant is first applied for hemostasis of the subchondral bone, followed by implant fixation with a second layer of fibrin sealant,^18^ or when multiple layers of fibrin sealant are applied within the lesion due to intraoperative displacement of the implant after initial fixation. The hypothesis was that the mechanical properties for both fibrin sealants are lower than what is desirable for its use as an adhesive in the fixation of cartilage fragments and implants.

## Methods

### Sample Preparation

Four juvenile bovine stifle (knee) joints were obtained from an abattoir (Research 87) within 48 hours of slaughter. Articular cartilage explants were obtained from the distal femoral condyles and trochlea of each joint. The regions from which samples originated were randomized for each test.

To test adhesion characteristics (pull-apart and lap shear), 5-mm-diameter articular cartilage and subchondral bone explants were taken. Cartilage explants were trimmed to 2 mm in thickness, retaining the superficial layer. Bone explants were also trimmed to 2 mm in thickness by removing the calcified cartilage layer and preserving the underlying trabecular bone with the subchondral plate left intact, as would be performed clinically to the chondral lesion bone bed.^19^

To test frictional properties (tribology), 10-mm-diameter explants were taken from the articular cartilage and trimmed to 5 mm in height. The bottom 2 mm of each explant was removed to create a disc and was retained. A 6-mm-diameter punch was used to create a ring-shaped explant (hereafter called an annulus) of native articular cartilage with the remaining 3-mm-thick tissue. The 2-mm-thick disc of cartilage was glued back to the bottom of the cartilage annulus using cyanoacrylate, taking care to not let the glue cover the interior of the cartilage composite. Additional cartilage was minced into cubes, approximately 1 mm^3^, to replicate particulated cartilage implants.

Fibrin sealants were stored at −20 °C, per the manufacturer’s recommendation, prior to use. Sealants were thawed at room temperature and used no later than 24 hours after thawing.

### Pull-Apart Tensile Testing and Lap Shear Testing

**Figure 1.**
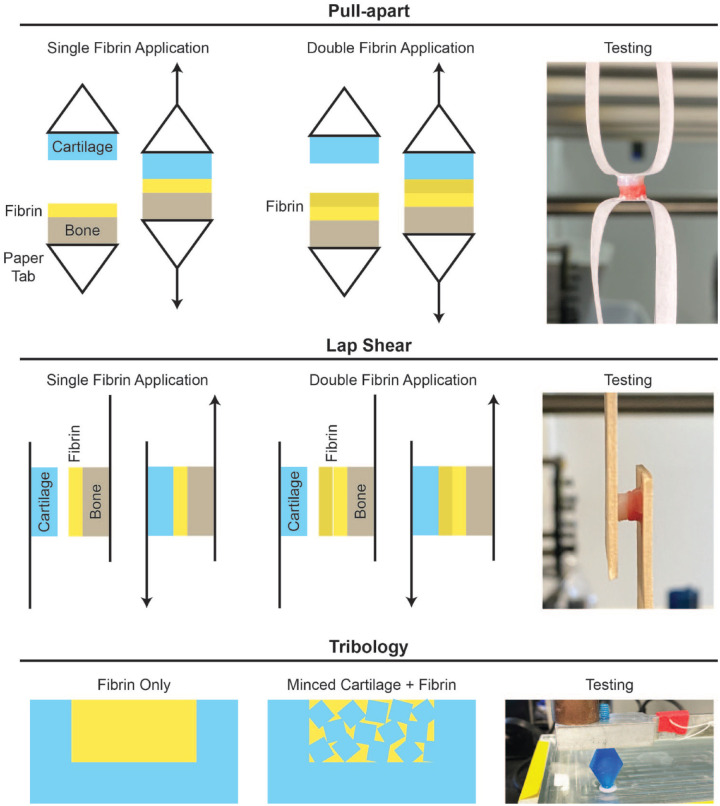
Testing setup.

For all pull-apart tests, the deep layer of the bone explants and the superficial layer of cartilage explants were each glued to paper tabs using cyanoacrylate. The paper tabs were fixed into an Instron 5565, such that their contact interface was oriented perpendicular to the applied tension (**Fig. 1**). All samples were tested in uniaxial tension at a constant rate of 1 mm/minute until sample failure. To analyze the resulting data, stress-strain curves were generated by normalizing force data to sample cross-sectional area. The adhesive stiffness was obtained from the slope of the linear region of the stress-strain curve, and the ultimate tensile strength (UTS) was defined as the maximum stress reached.

For all lap shear tests, the deep layer of the bone explants and the superficial layer of the cartilage explants were each glued to the middle of wooden tongue depressors, approximately 10 mm from the end, using cyanoacrylate (**Fig. 1**). All samples were tested in tension at a constant rate of 1 mm/minute until sample failure. To analyze the resulting data, stress-strain curves were generated by normalizing force data to sample cross-sectional area. The shear modulus was obtained from the slope of the linear region of the stress-strain curve, and the ultimate shear strength (USS) was defined as the maximum stress reached.

For testing of a single application (SA) of fibrin sealant, 10 µl of fibrinogen was pipetted onto the superior bone surface, immediately followed by 10 µl of thrombin. Immediately after the addition of thrombin, the inferior surface of the cartilage was placed directly on top of the bone and fibrin clot and held together with manual pressure for 2 minutes. After an additional 3 minutes without pressure, samples were tested.

For testing of a double application (DA) of fibrin sealant, 5 µl of fibrinogen and 5 µL of thrombin were pipetted onto the superior surface of the bone explant, as above. Using a moist, gloved finger, manual pressure was held on the clot and bone for 2 minutes. Then, an additional 5 µl of fibrinogen and 5 µl of thrombin were each added on top of the clot, and the inferior surface of the cartilage was placed directly on top. These composites were similarly held under manual pressure and tested as described for the SA composites.

For controls, a cartilage-bone composite was press-fit together under both dry and wet conditions. For dry controls, cartilage and bone explants were held together with manual pressure and no sealant for 2 minutes, followed by 3 minutes without pressure prior to testing. Wet controls were similarly tested but with the addition of 20 µl of saline to the bone surface prior to composite assembly.

### Tribological Testing

For minced cartilage + fibrin composites, approximately 10 pieces or 60 g of minced cartilage was placed into the cartilage annulus such that the level of minced cartilage reached the surface of the annulus. After adding 30 µl of fibrinogen and 30 µl of thrombin to immobilize the minced cartilage, a moist, gloved finger was used to apply manual pressure for 2 minutes to the composite, followed by 3 minutes without pressure prior to testing. For fibrin-only composites, 35 µl of fibrinogen and 35 µl of thrombin were added to the cartilage annulus and a moist, gloved finger was used to apply manual pressure for 2 minutes to the composite, followed by 3 minutes without pressure prior to sample loading in the tribometer. Tribology was performed using a custom-made pin-on-plate tribometer in phosphate-buffered saline under boundary lubrication conditions, with a velocity of 1 mm/second and a compressive normal force of 200*g*. The samples were allowed to equilibrate for 2 minutes, immersed in phosphate-buffered saline, and were then sheared against the glass test surface for 5 minutes (**Fig. 1**).

### Histology

Samples were fixed in 10% neutral buffered formalin and then decalcified in a 10% w/v ethylenediaminetetraacetic acid solution for 1 month. Following decalcification, sections were embedded in paraffin and sectioned to 8-µm-thick. Once mounted, sections were stained with hematoxylin and eosin to visualize cartilage, bone, and exogenous fibrin morphology.

### Statistics

A sample size of *n* = 4 per group was used for both phases based on the results of a power analysis (β = 0.8; α = 0.05) on preliminary data performed in JMP Pro 14 (SAS). In Phase 1, 4 groups were tested in tension and shear: a single application of Tisseel, a single application of Vistaseal, a dry press-fit control, and a wet press-fit control. For tribological testing, 4 groups were also tested: Tisseel alone, Vistaseal alone, Tisseel plus minced articular cartilage, and Vistaseal plus minced articular cartilage. Four samples (*n* = 4) were tested per group, yielding 16 samples per test, in total. For Phase 2, 2 groups were tested: a single application and a double application of each sealant. Four samples (*n* = 4) were tested per group, yielding 8 samples per test in total. All analyses were performed in Prism (GraphPad Software). Outliers were determined using a ROUT outlier test and were removed before further statistical analysis. A 1-way analysis of variance with Tukey’s multiple-comparisons post hoc tests was used to compare pull-apart, lap shear, and tribology data among experimental groups. An unpaired *t* test was used to compare pull-apart and lap shear data for SA versus DA of each fibrin sealant type. Data are presented as means with errors bars representing standard deviations. Groups marked by different letters are statistically different, with *P* < 0.05 indicating significance.

## Results

### Single Application of Fibrin Sealant

**Figure 2.**
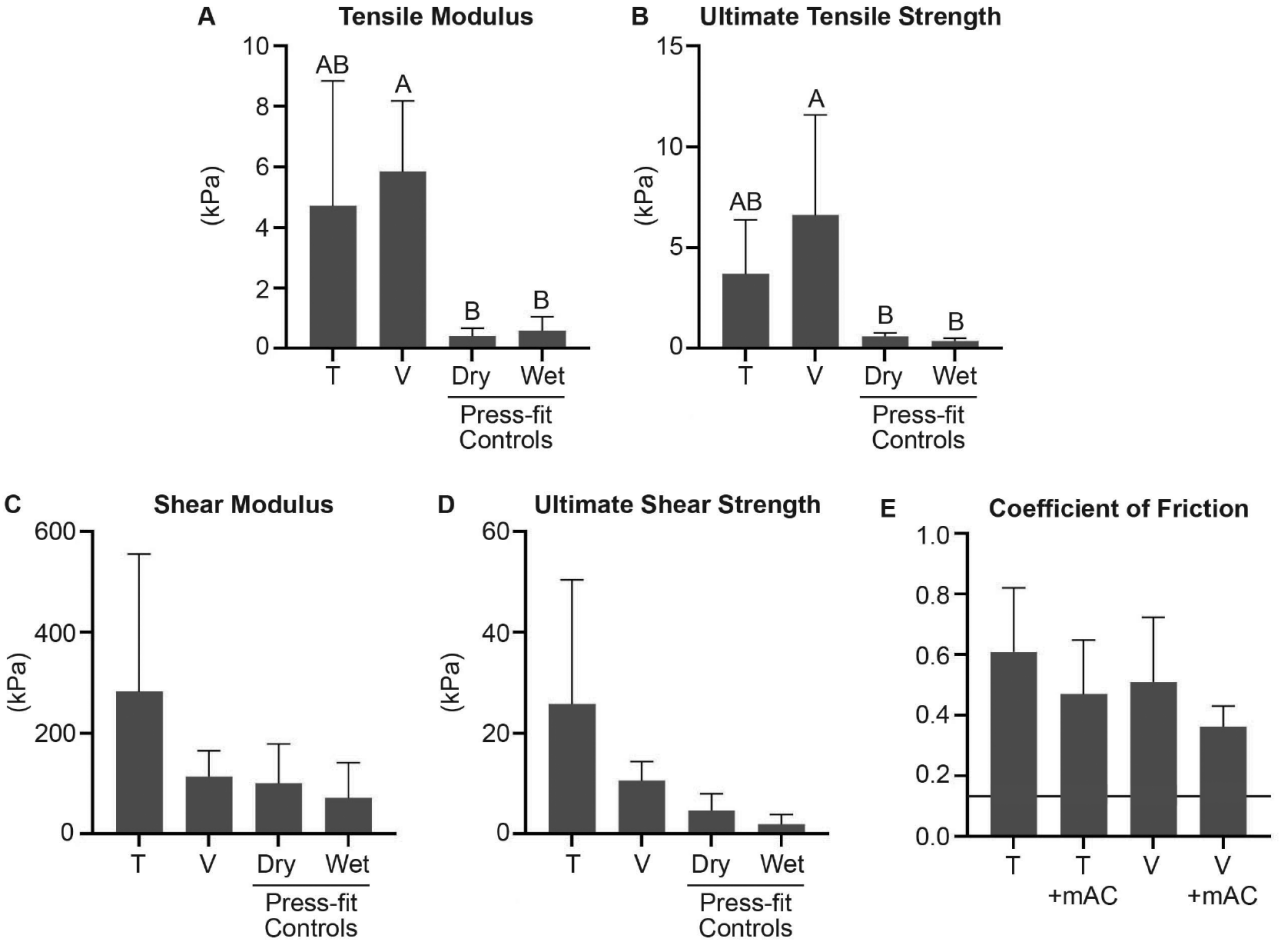
(A-D) Mechanical comparison of Tisseel (T) and Vistaseal (V), as well as dry and wet press-fit controls for tensile and shear properties. (E) For coefficient of friction, T alone and V alone were compared with minced articular cartilage (mAC) embedded in either T or V. Native cartilage coefficient of friction for bovine cartilage is represented by a horizontal black line.^26^ Statistical significance (*P* < 0.05) is indicated by groups marked with different letters.

Pull-apart testing revealed no difference in the tensile modulus or UTS between Tisseel and Vistaseal (**Fig. 2**; **Table 1**). The tensile modulus and UTS of Vistaseal were significantly greater than both press-fit controls. However, the UTS for both fibrin sealant types was <8 kPa. For reference, these values were several magnitudes lower than the 50-kPa UTS that is observed with application of cyanoacrylate adhesives (e.g., Dermabond) on the skin^20-23^ and far lower than the 4,400-kPa UTS that is observed with suture pull-out from bovine native articular cartilage.^24^ Similarly, the USS for both fibrin sealant types was <30 kPa, which is far lower than the 200 kPa observed with cyanoacrylate adhesives on the skin.^25^ There were no differences between Tisseel and Vistaseal for shear modulus, USS, or coefficient of friction (**Fig 2**; **Table 1**). In addition, there were no significant differences in the coefficient of friction when comparing Tisseel alone, Vistaseal alone, or minced articular cartilage embedded in either Tisseel or Vistaseal. The coefficient of friction for native bovine cartilage measured by pin-on-plate tribometry is presented in **Figure 2E** for reference.^26^

**Table 1.** *P* Values for Comparative Analyses.

Comparisons Among Tisseel, Vistaseal, and Controls	*P* Value
Pull-apart	
Tensile modulus	0.01
Tisseel vs. Vistaseal	0.91
Tisseel vs. dry control	0.10
Tisseel vs. wet control	0.12
Vistaseal vs. dry control	0.03
Vistaseal vs. wet control	0.04
Dry vs. wet control	1.00
Ultimate tensile stress (UTS)	0.03
Tisseel vs. Vistaseal	0.49
Tisseel vs. dry control	0.44
Tisseel vs. wet control	0.38
Vistaseal vs. dry control	0.05
Vistaseal vs. wet control	0.04
Dry vs. wet control	1.00
Lap shear	
Shear modulus	0.23
Ultimate shear stress (USS)	0.08
Tribology	
Coefficient of friction	0.31
Comparison between single and double applications	
Pull-apart	
Tensile modulus	0.82 (T), 0.16 (V)
UTS	0.35 (T), 0.63 (V)
Lap shear	
Shear modulus	0.41 (T), 0.95 (V)
USS	0.81 (T), 0.24 (V)

T = Tisseel; V = Vistaseal.

### Double Application of Fibrin Sealant

**Figure 3.**
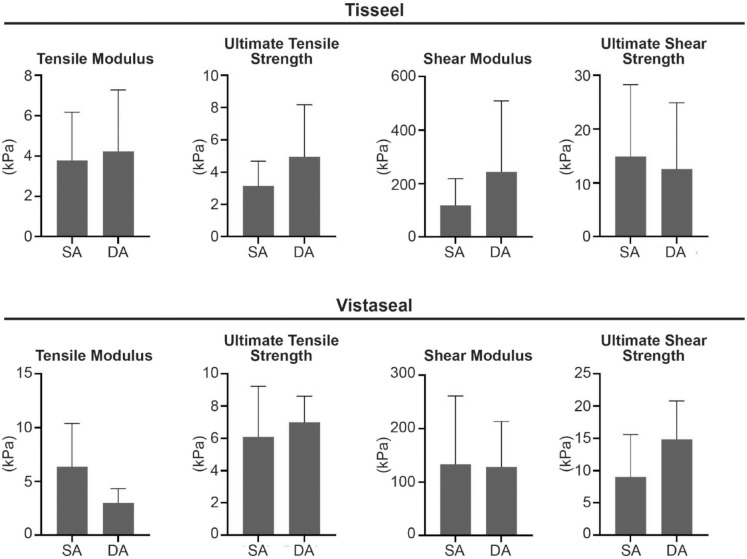
Mechanical comparison on single application (SA) and double application (DA) for both Tisseel and Vistaseal.

**Figure 4.**
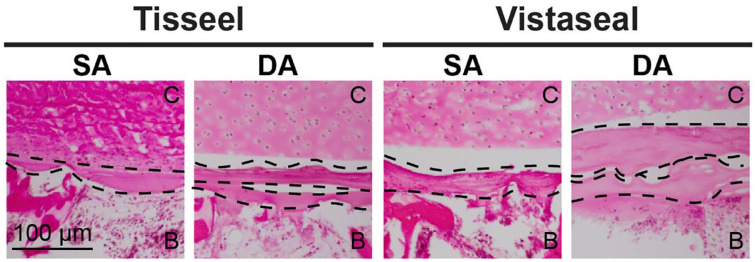
Histological comparison on single application (SA) and double application (DA) for both Tisseel and Vistaseal. Dotted lines delineate layers of fibrin. The cartilage layer is designated “C” and the bone layer is designated “B.”

There were no significant differences between SA and DA for both Tisseel and Vistaseal in tensile modulus, UTS, shear modulus, and USS (**Fig. 3**). Histology demonstrated that a DA of fibrin sealant resulted in 2 distinct and disconnected layers for both Tisseel and Vistaseal (**Fig. 4**).

## Discussion

In an *ex vivo* model for adherence of cartilage to bone, the objectives of this study were to (1) characterize the adhesive and frictional properties of 2 commercially available fibrin sealants and (2) determine whether there were differences in those properties based on fibrin sealant type or number of applications. Although the tensile modulus and UTS of Vistaseal were significantly greater than those of dry and wet controls, the adhesive properties for both fibrin sealants were much lower than what is desirable for clinical fixation of cartilage repair implants. No differences in adhesive and frictional properties were found between fibrin sealant types. Furthermore, there were no differences in mechanical properties between an SA and DA of sealant for both fibrin sealant types.

Fibrin sealant adhesion to various biologic tissues has been studied previously, but few have been evaluated for articular cartilage.^27-29^ A previous study using bovine articular cartilage composites demonstrated that both all-autologous fibrinogen/thrombin combinations provided similar cartilage-cartilage adhesion strength (15 and 25 kPa, respectively) as Tisseel (20 kPa).^30^ However, other commercial fibrin sealants and cartilage-to-bone adhesion were not explored in this study. The main difference between Tisseel and Vistaseal is the addition of aprotinin in Tisseel, which helps to slow down fibrinolysis. In this study, no differences in adhesion strength were observed between fibrin sealant types. It has been previously reported that fibrin clot strength may differ based on fibrinogen concentration.^30-32^ The differential amounts of fibrinogen present in Tisseel/Tissucol (Baxter Healthcare Corp, CA), Evicel/Quixil/Crosseal (Ethicon, Raritan, NJ) (60-80 mg), and Beriplast (CSL Behring, King of Prussia, PA) (90 mg) are suggested to be responsible for their varying adhesive strengths as demonstrated on non-cartilage tissues.^28,29^ The comparable fibrinogen concentrations in Tisseel and Vistaseal used in the present study may be responsible for their similar adhesive properties.^16,17^

The reduced adhesiveness of fibrin-to-cartilage compared to fibrin with other tissues, such as cardiovascular tissue, may be due to the extracellular matrix (ECM) properties of cartilage. Articular cartilage has a dense ECM with low permeability. These matrix properties may shield common fibrin(ogen) binding sites, such as fibronectin and vitronectin, from fibrin(ogen) during clot formation.^33^ It has been suggested that the stronger cartilage-fibrin adhesion achieved with platelet-poor plasma–derived fibrinogen compared with platelet-rich plasma (PRP)-derived fibrinogen was due to a slower clotting time, which allowed for deeper penetration of the fibrinogen into the cartilage ECM before fibrin clot formation.^30^ PRP is generated from the heavier layers of blood that has been centrifuged and has higher concentration of fibrinogen compared with PRP, which is generated from the centrifugal waste by-product.^30^ In addition, like the ECM of cartilage, fibrinogen is negatively charged.^34,35^ Therefore, repulsive forces of the 2 negative charges may also impede fibrin(ogen) permeation into and binding with the cartilage ECM. Given the need for early joint motion postoperatively to provide nutrition to the cartilage repair via movement of synovial fluid and prevent arthrofibrosis,^36^ cartilage adhesion with fibrin sealant should be much stronger than the properties demonstrated in this study and raises concern for implant displacement in the postoperative period.

This is the first study to examine the tribological properties of fibrin sealants in the context of cartilage repair. The frictional properties were not different between fibrin sealant type. Furthermore, the coefficients of friction were not significantly affected when minced articular cartilage was added to the defect model, implying that the increased friction is primarily due to fibrin, not the minced cartilage. Any fibrin sealant applied to the native cartilage implant periphery would be expected to provide little adhesion or frictional forces due to the low surface area at the periphery. Although the coefficients of friction were greater than what has been reported for native bovine articular cartilage (0.13),^26^ the resultant peripheral friction from fibrin sealant is not expected to provide substantial resistance to tensile or shear displacement of the implant *in vivo*.

Number of fibrin sealant applications did not yield significant differences in their mechanical properties. Because excessive bleeding at the bone base can cause implant displacement and chondrocyte apoptosis, some surgeons prefer to apply an initial layer of fibrin sealant on the subchondral defect bed to achieve hemostasis prior to fixation of a cartilage implant.^18^ DA of fibrin sealant can also occur when the cartilage implant displaces intraoperatively after the initial application, and the implant is refixed with a second application of fibrin sealant to the defect bed. Histologically, DA of both sealants resulted in 2 distinct and noncontiguous layers of fibrin. However, this did not reduce the adhesion properties of the cartilage-bone composites. These results suggest that multiple applications of fibrin sealant on the subchondral bone prior to cartilage implantation are neither detrimental nor beneficial to the adhesion of the implant.

There were several limitations to this study. Testing was performed with native cartilage and bone explants rather than commercially available cartilage repair products, such as MACI or DeNovo NT. Therefore, the model only replicates the clinical scenario of repairing displaced autologous cartilage fragments, and mechanical properties may differ when fibrin sealant is applied to the porcine collagen membrane of MACI. In addition, the explant composite model did not replicate a defect with surrounding peripheral cartilage. However, as aforementioned, this periphery provides little resistance to forces that would dislodge an implant. The cartilage-bone composite model was selected for this study to allow for standardization and enable a more accurate measurement of resistance to tension and shear displacement. In addition, this study was performed without synovial fluid. The presence of synovial fluid has been shown to decrease the mechanical stiffness of the final clot up to 90% and increase fluid permeability of the clot by up to 468-fold.^37^ While not performed in the presence of synovial fluid, the results of this study still hold clinical weight because cartilage implantation techniques include hemostasis and drying of the defect bed prior to fibrin sealant application. While the sample number used for the tests in this study was determined via a power analysis of preliminary data, the total number of samples was relatively low. Thus, future studies should aim to determine whether differences in Tisseel and Vistaseal are elucidated based on other testing modalities, such as torsional mechanical testing or testing in the presence of synovial fluid. Finally, the minimum adhesion strength required to retain cartilage implants *in vivo* is unknown and warrants further study. Given that modified acrylates, such as Dermabond, yield clinical outcomes comparable to suturing of the skin,^38^ cartilage-specific adhesives should exhibit adhesive bonding strength of at least 50-200 kPa.

This study characterized the mechanical properties of commonly used fibrin sealants for adhering cartilage to subchondral bone in the context of cartilage repair. It was shown that the adhesion and frictional properties are similar between Tisseel and Vistaseal. However, the adhesive properties of these fibrin sealants for cartilage-to-bone were lower than what may be desired to retain cartilage implants *in vivo*. These findings motivate the development of a cartilage-specific adhesive that promotes stronger cartilage implant fixation, such as those that crosslink the collagen present in native cartilage and cartilage implants. Additional research is being performed toward the development of cartilage adhesives based on gelatin, albumin, and naturally occurring adhesives generated from mussels and barnacles.^39^ However, the issue of cartilage implant fixation and integration continues to be a prominent hurdle impeding clinical outcomes for cartilage repair surgery.
